# Construction of Epstein-Bar virus cocktail peptide fused with Fcγ of IgG: as a potential delivery system for vaccine development

**DOI:** 10.1080/21655979.2019.1694388

**Published:** 2019-11-25

**Authors:** Saeid Amel Jamehdar, Samira Tabaei, Baratali Mashkani, Reza Karimi, Morteza Motallebnezhad, Arezoo Esmaili

**Affiliations:** a Antimicrobial Resistance Research Center, Mashhad University of Medical Science, Mashhad, Iran; b Department of Immunology, School of Medicine, Shiraz University of Medical Sciences, Shiraz, Iran; c Department of Medical Biochemistry, School of Medicine, Mashhad University of Medical Sciences, Mashhad, Iran; d Department of Biology, Damghan Branch, Islamic Azad University, Damghan, Iran; e Immunology Research Center, Tabriz University of Medical Sciences, Tabriz, Iran; f Student Research Committee, Tabriz University of Medical Sciences, Tabriz, Iran; g Department of Immunology, Faculty of Medicine, Iran University of Medical Sciences, Tehran, Iran

**Keywords:** Epstein-Bar virus, *Pichia pastoris*, peptide vaccine, fusion peptide

## Abstract

Epstein–Barr virus (EBV) associated with several diseases such as contagious mononucleosis chronic active EBV infection, and diverse sorts of malignant tumors. Therefore, using applicable vaccines could be advantageous for public health. Yet, the vaccine has been unavailable to protect from EBV so far. In the current study, to develop a multi-peptide vaccine for EBV and assess its expression in *Pichia pastoris* yeast system, three immunodominant sequences in glycoprotein (gp) 85, gp350 and latent membrane protein 1 (LMP1) were chosen. To construct fusion peptide, -GGGGS- liker was applied. After cloning the fusion peptide in the pPICZαA expression vector, this recombinant vector processed and transfected into *Pichia pastoris* host cells. The expression of high level of EBV fusion peptide was confirmed by dot blot and SDS-PAGE procedures. The *Pichia pastoris* is capable of supporting EBV fusion peptide expression. The application of this fusion peptide as a peptide vaccine to fight EBV is suggested.

## Introduction

It is believed that there is a link between Epstein-Barr virus (EBV) and various human malignancies [i.e. nasopharyngeal carcinoma [NPC], Burkitt and Hodgkin lymphoma] in immunocompetent and immunosuppressed people. The emergence of primary infection is normally observed in the first few years of life and it is typically without symptoms. A study reported 70% of Iranian and 56% of German 1–5 year-olds were infected with EBV [1,2]. It has been argued that 95% of the adolescents are persistently infected leading to other more serious disease states such as B cell lymphomas, nasopharyngeal carcinoma, and gastric carcinoma [3,4]. The link between gastric carcinomas and EBV has been reported and it is believed that 90% of gastric lymphoepitheliomas are EBV positive [5]. Therefore, developing therapies to decrease mortality from the disease and suffering from the treatment seems essential [6]. It is necessary to develop vaccines in a way that are capable of either blocking the primary EBV infection or considerably decreasing the EBV load during primary EBV infection. Since an effective cytotoxic T cell (CTL) response is not elicited by the immunization with completely viral proteins, researchers are trying to develop peptide vaccines according to the defined epitope sequences [7]. A membrane consisted of four major virus-specific proteins, namely, gp350, gp220, gp85 covers the EBV. The focus of the majority of methods to develop EBV vaccines was the virus membrane antigen; it consists of at least three glycoproteins [7]. The most abundant glycoprotein on the surface of virions, the EBV gp350, has been favored because it is capable of eliciting CD4^+^ T-cell responses and neutralizing this virus. Thus, researchers have focused on the probable targeting of the virus-specific immune response to the viral antigens, which are expressed in these malignancies [8]. According to the previous findings, allow CTL precursor frequency to epitopes within LMP1 (an integral membrane protein) in healthy virus carriers; thus, it is likely that the long-term therapeutic benefit in patients with NPC requires the re-formation of both LMP1-specific and LMP2-specific CTL responses [9]. The ability of the vaccines to induce protective immune responses against viral infection is limited by reaching effective uptake by antigen-presenting cells (APCs). One of the methods to tackle this problem is using the Fc-fusion proteins in which one or more antigenic proteins are fused into the Fc domain of immunoglobulin [10]. Besides, the fusion of immunodominant proteins to the Fc domain is capable of increasing harvesting and delivering them to the T lymphocyte, which is of tremendous importance for protection against EBV [11]. To date, there has been no effective vaccine for passive immunization against the virus yet. Due to *Pichia pastoris* superiority than other expression systems for the production of an active functional recombinant vaccine. In this study, we expressed a fusion peptide consisting of gp350, gp85, and LMP-1:Fcγ2a and Fc domain of mouse IgG2a in *Pichia pastoris* (*P. pastoris*) system as a novel probability approach to design vaccine against EBV.

## Material and methods

### Strains, plasmids, enzymes, and cell line

T-cell epitopes for EBV proteins were chosen through a literature review. These epitopes were Gp350_863-871,_ Gp350_147-165_ [3] (Gen Bank access number GI: 82,503,223), gp85_225-233_, gp85_420-428_, gp85_542-550_ (Gen Bank access number GI: 557,791,647) and Lmp1_161-169_ (Gen Bank access number GI: 23,893,668) [12]. To combine these epitopes, a universal -GGGGS- liker was used. Lastly, the Fc fragment of mouse IgG2a (Gen Bank access number GI: 51,835) [13] was fused to the C-terminal of fusion epitopes. DNA 2.0, Genescript and Genius software’s were used to study the codon-optimized and colon simulating in *Pichia pastoris*.

Fusion peptide EBV flanked by XhoI/XbaI restriction enzymes. pPICZαA expression vector carries the α-factor signal sequence (α-MF) to drive fusionic peptide secretion. Moreover, this vector carries the zeocin resistance gene, which can be used to choose *E*. coli and *Pichia pastoris* recombinants; a termination signal was formed by adding one stop codon at the C terminus to form. c-myc was applied in vector design for the detection of the fusion peptide by dot blot assay. This designed peptide sequence was ordered in pGH vector by Nrdayefan company, Iran.

### Strains and media

Escherichia coli JM109 strain was applied as a host for cloning and manipulating manipulation. This strain was cultured at 37°C in Luria-Bertani (LB) broth medium supplied with ampicillin. The *P. pastoris* strain GS115 (Invitrogen, CA, USA) and the expression vector pPICZαA were used as the expression hosts. The yeast cells were grown at 30°C on Yeast Extract–Peptone–Dextrose (YPD) Medium (1% yeast extract, 2% Bacto-peptone 2% glucose) and Yeast Extract–Peptone–Dextrose- sorbitol (YPDs) Medium (1% yeast extract, 2% peptone, 2% glucose, 1 M sorbitol) supplemented with 100 μg/mL zeocin if necessary. Restriction enzymes used for cloning were bought from Promega (Madison, Wisconsin, USA) and applied following manufacturer’s recommendations.

### Preparation of fusion peptide sequence

To amplify this synthetic fusion sequences, the pGH vector was transformed into JM109 E.coli strain through calcium chloride method. Plasmid was extracted by the commercial kit (Bioneer, Korea). Double digestion with related restriction enzymes and sequencing verified the presence of the target segment.

### Construction of ppiczαa- gp350-gp85-Lmp1-Fc

Following the minipreparation of pGH containing fusion sequence and double digestion, the target segment was gel extracted and purified by commercial kit following the manufacturer’s instructions (5 prime, Germany). This segment was cloned into pPICZαA shuttle vector controlled by AOX1 promoter that led to pPICZαA- gp350-gp85-Lmp1-Fc. The restriction digestion, polymerase chain reaction (PCR) amplified entire recombinant fusion peptide sequence were used to verify the construction of the vector. The primers 3´AOX1, 5´-GCAAATGGCATTCTGACATCC-3´ and α-Factor, 5´-TACTATTGCCAGCATTGCTGC-3´ were applied to implement PCR. PCR was carried out by 10 pmol of each primers and PCR mix (5 prime, Germany). All primers were purchased from Sina gene, Iran. The temperature profile was one cycle in 94°C for 3 min followed by 30 cycles in 94^°^C for 1 min, 55^°^C for 1 min, and 72^°^C for 2.30 min and finally 72^°^C for 1 min (Bio-rad MyCycler Thermal Cycler, USA).

### Electroporation of yeasts

The recombinant vector pPICZαA–gp350-gp85-Lmp1-Fc was linearized with SacI was used to linearize the recombinant vector first; then, phenol-chloroform was used to purify it; at last, it was electroporated into competent *P. pastoris* GS115 cells (Bio-Rad, Gene Pulser Xcell™ Electroporation Systems, USA) that is controlled by the AOX1 promoter. The phenol-chloroform-propanol alcohol was used to purify this recombinant plasmid. YPD plates containing 0.1 mg/mL Zeocin were used to screen pPICZαA–gp350-gp85-Lmp1-Fc -positive transformants; then, the recombinant *p. pastoris* cells were subjected to BMGY for fusion peptide expression. The positive transformants resistant to 0.1 mg/mL Zeocin were inoculated into 10 mL buffered complex glycerol media (BMGY) (containing 1% yeast extract, 2% peptone, 100mM Potassium phosphate pH 6, 1.34% yeast nitrogen base, 4 x 10–5% biotin and 1% glycerol). Vigorous shaking (300 rpm) at 30°C until the culture reached OD600 = 1.5 (Double Beam Uv Vis Spectrophotometer 2377, India) was applied for eliminating the inhibitory effect of other carbon sources. For inducing AOX1, the above product was put into the BMMY medium and incubation was done at 30°C. In this system, %0.5 methanol was used as the inducer that was added into the medium from day 2 up to day 7. A negative control containing the empty pPICZαA vector was performed likewise. Dot blot and sodium dodecyl sulfate-polyacrylamide gel electrophoresis (SDS-PAGE) (Bio-Rad, USA), were applied to verify the expression of gp350-gp85-Lmp1-Fc.

## Result

### Bioinformatics analysis

**Figure 1.**
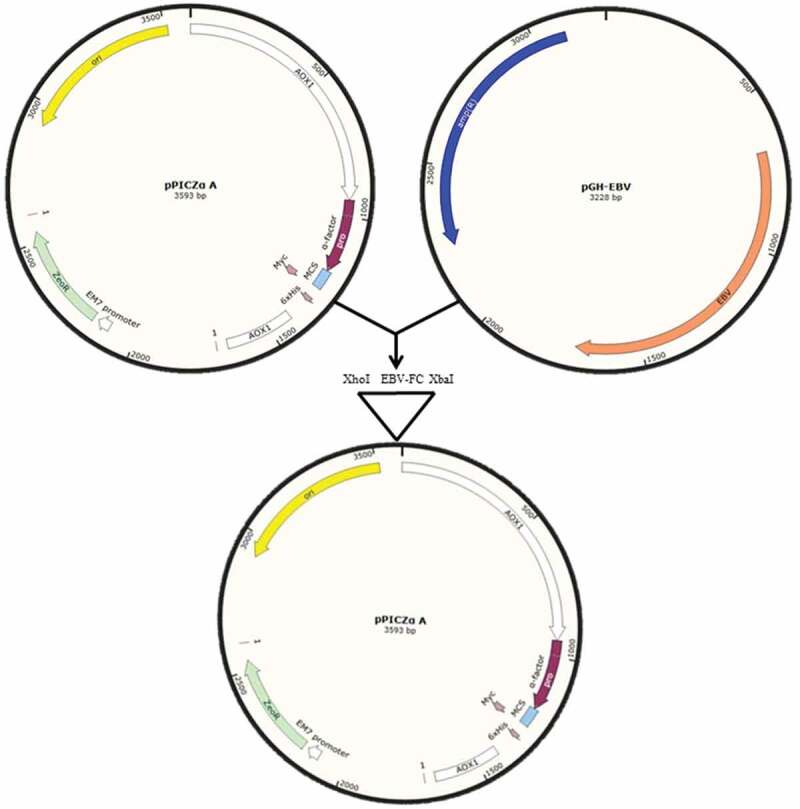
Schematic diagram of cloning modified EBV segments in *XhoI/XbaI* site of MCS of pPICZαA. (pPICZαA-EBV).

Alignment of the gp350-gp85-Lmp1-Fc amino acid sequence before and after the optimization indicated a high amino acid sequence resemblance after optimization. In the present study, pPICZαA and *P. pastoris* strain GS115 were employed as an expression vector and host cell, respectively. α-factor from *S. cerevisiae* was codon-optimized to drive the secretion of fusion peptide. For the expression the protein, the optimized gp350-gp85-Lmp1-Fc segments were then inserted into the MCS (multiple cloning site) from plasmid pPICZαA, ligation was amplified with T4 DNA ligase, selected as pPICZαA–gp350-gp85-Lmp1-Fc with XhoI/XbaI enzymes (Figure 1).

### Construction and transformation of ppiczαa – gp350-gp85-Lmp1-Fc

**Figure 2.**
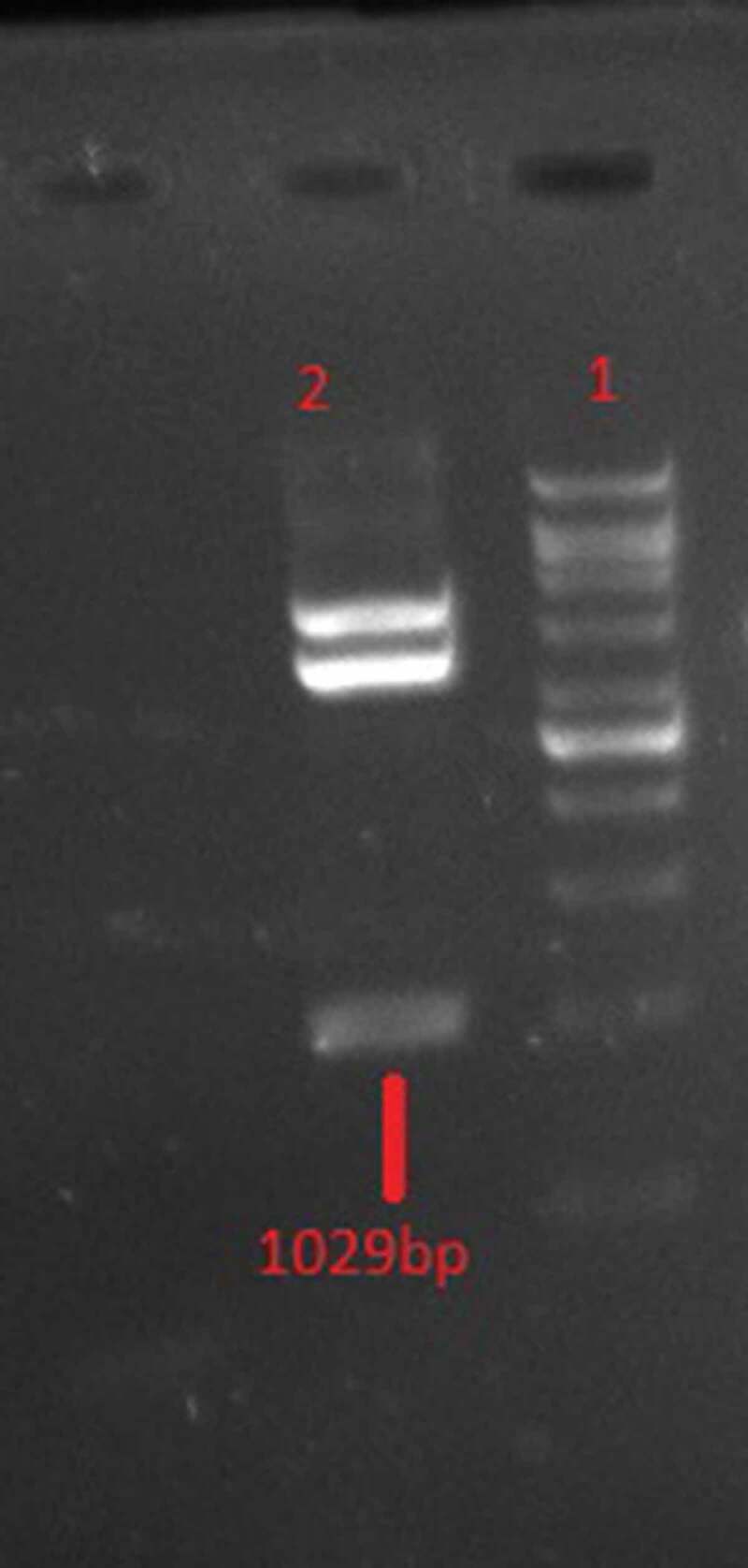
Double restriction digestion analysis of pPICZαA-EBV with *XhoI/XbaI* restriction enzymes. Line1: 1Kb DNA Ladder, Line 2 digested recombinant plasmid.

**Figure 3.**
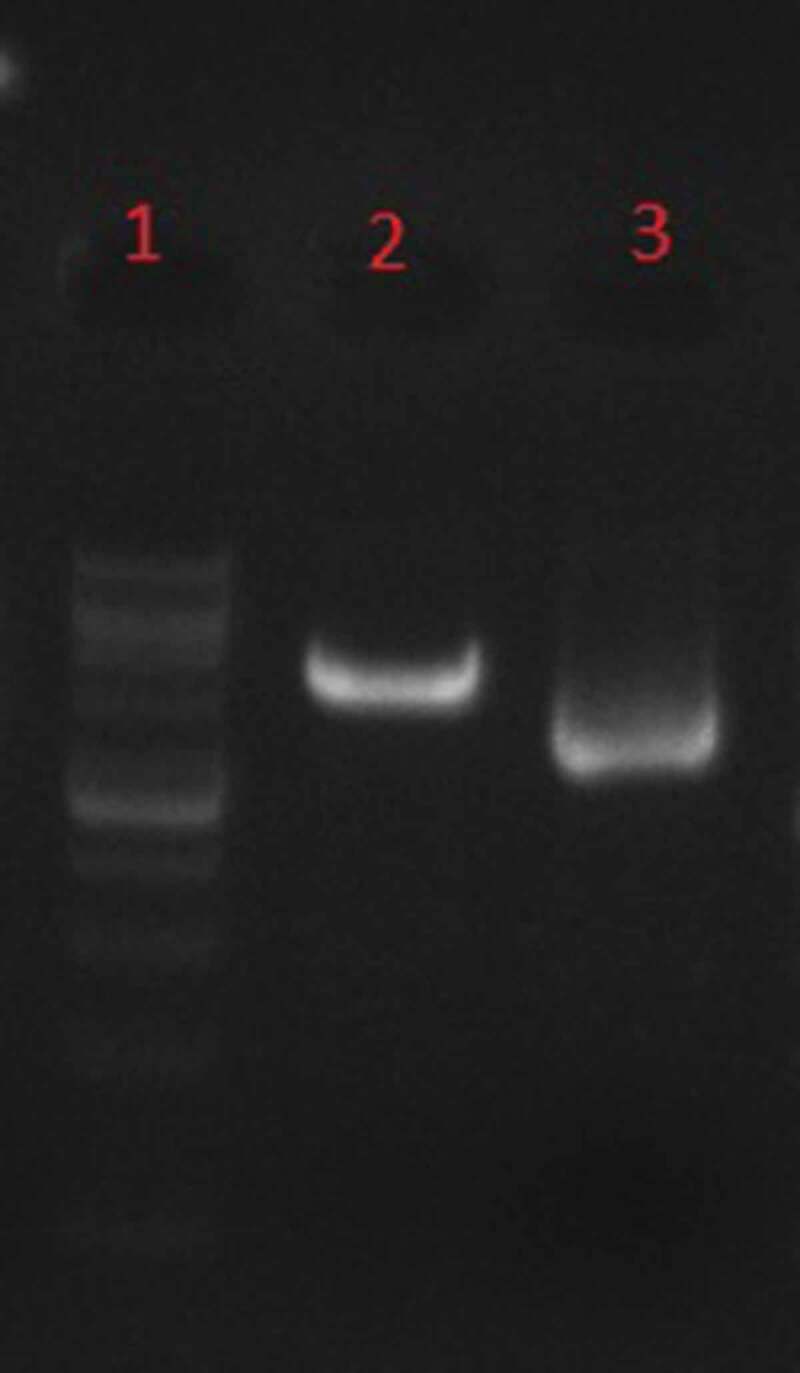
Phenol–chloroform extraction, Line1: 1Kb DNA Ladder, line 2: linearized recombinant plasmid by SacI, line 3: undigested recombinant plasmid.

According to double digestion with XhoI/XbaI enzymes (Figure 2), there is a ligation between gp350-gp85-Lmp1-Fc segments and pPICZαA expression vector. A fragment with the expected length of ~1030 bp was produced by the PCR and restriction digestion of the recombinant plasmid pPICZαA- gp350-gp85-Lmp1-Fc. DNA fragments with the expected length of the target gene 1030 bp were produced in the transformed yeast clones. As shown in Figure 3, SacI was used to linearize the results of a recombinant plasmid, pPICZαA- gp350-gp85-Lmp1-Fc that could be integrated by homologous recombination into the AOXI region *P. pastoris* GS115 cells by electroporation.

### *Expression of ppiczαa- gp350-gp85-Lmp1-Fc in* pichia pastoris

**Figure 4.**
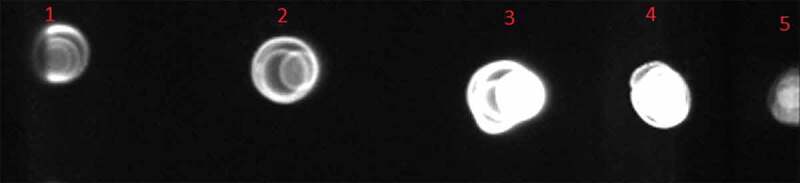
Dot blot results. Fusion peptide was present in the supernatant during total course of cultivation (7 days).

**Figure 5.**
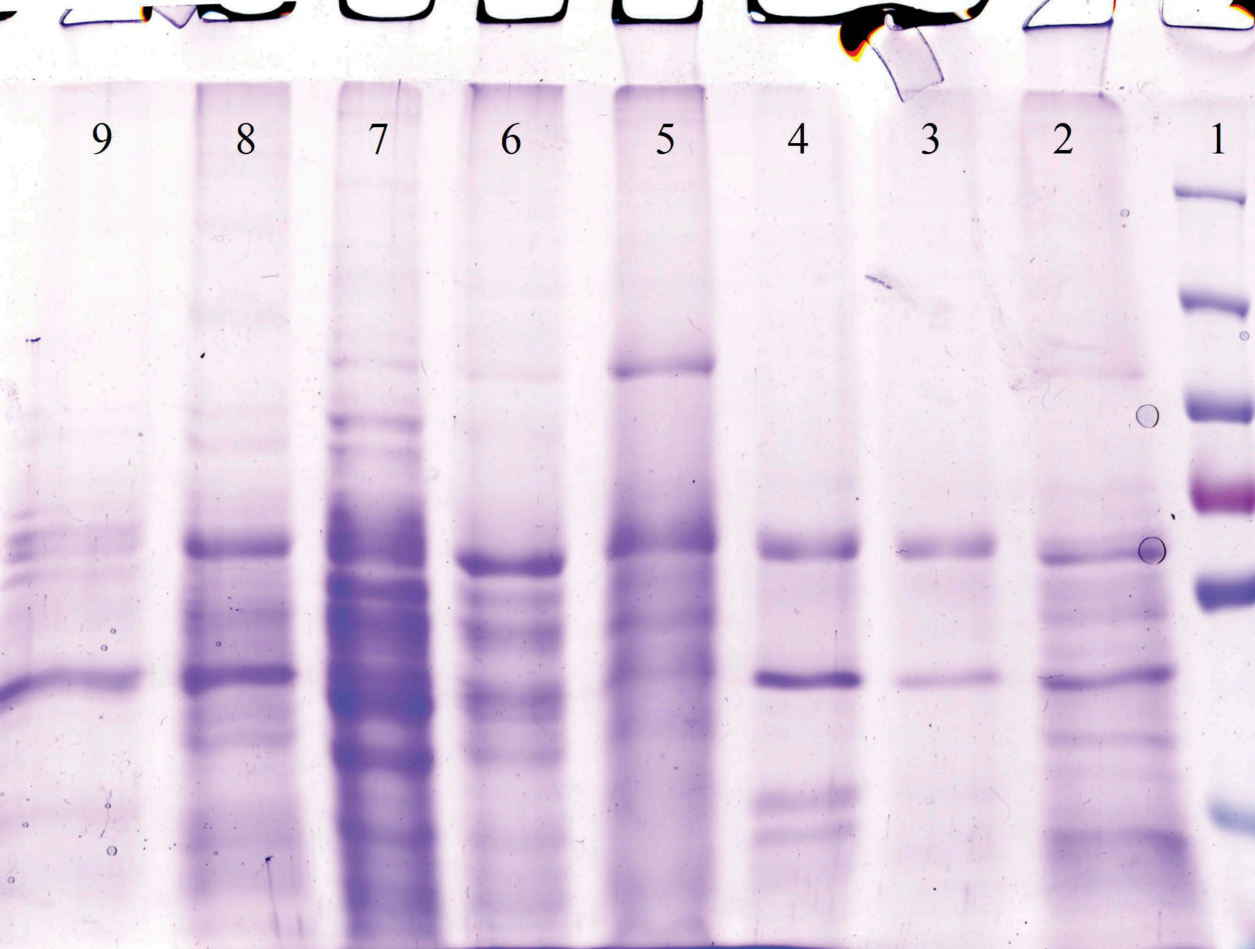
The SDS-PAGE of the *P. pastoris* GS115/pPICZαA-EBV cells revealed a protein band profile with a molecular mass of ~50 kDa. EBV protein was not found in the *P. pastoris* GS115/pPICZαA cells. Line 1: Protein ladder, Line 2–4: pPICZαA with EBV fusion peptides and Line 5: pPICZαA without fusion peptide.

Methanol was used to induce the *P. pastoris* GS115 cells containing pPICZαA – gp350-gp85-Lmp1-Fc. The recombinant fusion protein was release to BMGY media. The protein band was approximately 50 kDa in the supernatant of four positive transformants (pPICZαA – gp350-gp85-Lmp1-Fc). Dot blot and 10% SDS-PAGE were used to explore the supernatant of the media after pPICZαA- gp350-gp85-Lmp1-Fc was expressed in the induction media. In dot blot, antibody was used against c-myc epitopes in terminal segment of the cassette gene and confirm that fusion EBV peptide was present in media yeast culture supernatant (Figure 4). SDS-PAGE to confirm the expression of gp350-gp85-Lmp1-Fc protein. In SDS-PAGE, coomassie blue staining indicated the production of a fusion peptide by three of four induced clones; this fusion peptide was not present in the control strain its molecular weight was according to our expectations (~50 kDa) for gp350-gp85-Lmp1-Fc (Figure 5).

## Discussion

Various studies have emphasized the involvement of the immune system in the defense against EBV-associated diseases. Many studies reported that gp350, gp85, and lmp1 is a significant target for both CD4^+^ and CD8^+^ T cell responses [3,10,14]. Yet, no specific treatment/vaccine has been introduced against EBV infection [15].

In the present study, six immunodominant epitopes were chosen on three immunologic proteins gp350, gp85, and LMP1 of EBV virus and FC and fused by -GGGGS- linkers. According to our findings, a yeast system can be used for the expression of the fusion peptide EBV can be expressed in a yeast system by dot blot and SDS-PAGE technique. It has been shown that developing virus-speciﬁc CD8^+^ and CD4^+^T cells necessitates long-term protection from persistent viral infection besides the natural killer (NK) cells that are capable of identifying viral Ags in association with either class I or class II MHC molecules. Since immunization with whole viral proteins is not capable of eliciting an effective CTL response, researchers are trying to design vaccines based on deﬁned epitope sequences [10]. To stop infection or IM limited number of EBV vaccine trials have been done in humans [14]. In the past, researchers tried to find a vaccine that was able to stop EBV infection and decrease the incidence of EBV-associated malignancies.

Nevertheless, developing such a vaccine would mean tackling some problems. Apart from the information obtained from experiments with vaccines for other herpesvirus, indicating that it is unlikely that a primary EBV infection could be entirely prevented through the use of a vaccine, limited experimental information may be obtained regarding the efficacy of a possible vaccine since EBV only infects human [14]. Detecting the possible CTL determinants within EBV structural proteins is very important because that immunization with whole viral proteins is not capable of eliciting an effective CTL response. Furthermore, a vaccine based on CTL epitopes would cover determinants not only from gp350 but also from other structural Ags, such as gp85. To deal with this issue, a new protocol was used in the present study to detect CTL epitopes within gp350 and gp85 [12]. Some vaccines play a protective role even when the neutralizing antibody is not present, thus that gp350 vaccines induce antibodies with other activities, such as antibody-dependent cellular cytotoxicity or T-cell responses that are protective [5]. Wang *et al*. produced recombinant EBNA1 protein in Pichia pastoris and assessed its immunogenicity. Besides, mice immunized with E1ΔGA developed CD4^+^ and CD8^+^ T cell responses. According to these results, the yeast-expressed E1ΔGA reserved good immunogenicity and it could be a vaccine candidate against EBV-associated malignancies [16]. Placebo-controlled clinical trials have been done to develop and assess two very different EBV vaccines with adjuvants. One was a gp350 subunit antigen and the other was a CD8^+^ T-cell peptide epitope. Nevertheless, investigations on a gp350 vaccine have not been stopped, since they are trying to prevent primary EBV infection or to stop disease progression [17]. Cohen *et al*. in the only phase 2 trial of an EBV vaccine in humans, soluble gp350 in alum and monophosphoryl lipid an adjuvant reduced the rate of infectious mononucleosis in EBV seronegative adults but did not affect the rate of EBV infection [12]. In the present study, two immunodominant peptides were chosen, namely, QNPVYLIPETVPYIKWDNC (147–165) and VLQWASLAV (863–871) in gp350. Seemingly EBV gp350 is a valid immunogen for a vaccine to stop IM, but whether a vaccine with additional EBV antigens could effectively prevent virus infection remain unknown [12]. EBV proteins expressed in these malignancies (EBV latent membrane proteins 1 and 2 [LMP1, LMP2] are the probable options [14]. Today, a dendritic cell vaccine targeting LMP1 and LMP2 (expressed in NPC) successfully went through a phase II trial of immunotherapy in EBV-positive metastatic NPC patients; however, its effectiveness is restricted [14]. Consequently, LMP1 and LMP2 are the only available target antigens that can be used for developing new strategies for increasing antigen-speciﬁc T-cell immunity to treat HD and NPC [18]. In the present study, an immunodominant peptide, (161–169) YLQQNWWTL in LMP1 was chosen.

According to the current studies, gp85 can be a potential target for vaccine design. Burrows *et al*. were able to detect CTL epitopes within the EBV structural antigen gp85. An animal model system further indicated that gp85 epitopes could generate structural antigen-specific CTL responses and decrease infections with the virus expressing gp85. A vaccine including some CTL epitopes that protects more than 90% of the Caucasian population has been developed by Queensland Institute of Medical Research [6]. In the present study, three immunodominant peptides, namely, SLVIVTTFV (225–233), TLFIGSHVV (420–428) and LMIIPLINV (542–550) on gp85 were selected. One of the main limitations for applying subunit vaccines for inducing protective immune responses against viral infection is the effective uptake by antigen-presenting cells (APCs). This issue can be tackled using the Fc-fusion proteins where one or more antigenic peptides are fused into the Fc domain of immunoglobulin [10]. According to earlier investigations, the effectiveness of antigen uptake and presentation by these cells can be improved roughly 50–500 fold upon the targeting of antigens to FcγR on APCs, in the form of fusion with Fc domain [19]. In the present study, the selected peptides were fused selected peptides to mouse IGg2A Fc. Still, fusing immunodominant peptides to Fc domain is capable of increasing harvesting and delivering them to the T lymphocyte, which is very important for EBV protection [11]. The expression system of *P. pastoris* has some positive features including low cost, ease of genetic manipulation, stable expression, and rapid growth rate [13]. It is possible to induce foreign genes in *P. pastoris* to a high level of expression, and the expressed proteins could be subjected to post-translational modifications that are crucial in the recombinant expression of eukaryotic proteins [20]. Meanwhile, only small amounts of endogenous proteins are secreted by *P. pastoris*, thus, secreted recombinant protein would be the main protein in the medium making the purification process easier [16,21]. In a similar study on Cytomegalovirus (CMV), Tabaei *et al*. showed that fusion CMV peptide can be produced at appropriate levels by *P. pastoris* [8]. This result conflicts with our findings. According to the findings of the present study, *P. pastoris* yeast was used successfully as an expression system to generate EBV fusion peptide, which is capable of secreting it to the culture medium. The aim of the present study was reaching a high-level expression of EBV fusion protein in *P. pastoris* to develop beneficial peptide vaccines. Immunogenicity studies are needed to be carried out to clearly find out the role of these fusion peptides for candidate vaccine against EBV.

## Conclusions

According to our findings, it is possible to produce the gp350-gp85-Lmp1-Fc at appropriate levels by *P. pastoris*. The present study has paved the way for future immunological studies on this vaccine. We believe downstream processing could be supported by the secretion of EBV gp350-gp85-Lmp1-Fc in the culture media. Further experiments are required for specifying the immunogenicity of this cocktail peptide, but the data presented here show that this vaccine could be potentially used to develop a new peptide vaccine.

## Highlights

- Epstein–Barr virus (EBV) associated with several diseases.
- Yet, the vaccine has been unavailable to protect from EBV so far.
- The *Pichia pastoris* is capable of supporting EBV fusion peptide expression.
- The application of EBV fusion peptide as a peptide vaccine to fight EBV is suggested.
